# A Case of Recurrent Embolic Strokes in a Young Female With a Patent Foramen Ovale and Presumed Fibroelastoma

**DOI:** 10.7759/cureus.26722

**Published:** 2022-07-10

**Authors:** Nardine Abdelsayed, Kevin Parza, Mohamed Faris

**Affiliations:** 1 Department of Internal Medicine, Grand Strand Medical Center, Myrtle Beach, USA

**Keywords:** rope index, protein s deficiency, lupus anticoagulant, cerebrovascular accident, fibroelastoma, stroke, patent foramen ovale

## Abstract

Patent foramen ovale (PFO) occurs in about 25% of the population. PFO closure has been shown to decrease the risk of stroke in patients with recurrent strokes as compared to those treated with medical therapy alone, with more benefit in those with larger PFO sizes. Percutaneous PFO closure, although minimally invasive, does carry surgical risks, which must also be taken into account. We present a case of a 31-year-old female presenting with a left middle cerebellar artery (MCA) stroke and persistent deficits who was found to have both a PFO and presumed fibroelastoma on her aortic valve. She was treated with aspirin and apixaban and advised to follow up with cardiothoracic surgery once she recovered from her stroke for ultimate PFO closure and removal of the fibroelastoma. Unfortunately, she presented again less than one month later with recurrent cerebrovascular accidents (CVA) requiring urgent PFO closure. Our case stresses the importance of tools such as the Risk of Paradoxical Embolism (RoPE) score index when determining treatment plans for patients with PFO, and possible confounding factors such as the presence of an aortic valve fibroelastoma.

## Introduction

Cerebrovascular accidents (CVA) in young patients are most commonly of cardioembolic origin. Examples include anatomic abnormalities such as patent foramen ovale (PFO) and atrial septal defects (ASD). PFOs occur when the foramen ovale fails to close in a fetus, resulting in an abnormal connection between the left and right atrium. They are quite common in the general population [1] and are generally asymptomatic except in a segment of the population that develop CVA or severe migraines. The mechanism of CVA in PFOs and ASDs is most commonly due to "paradoxical embolism", which occurs when a venous thrombus travels to the right atrium and passes through the PFO or ASD to the left atrium, ultimately embolizing to the cerebral circulation. PFO closure has been found to decrease the risk of stroke in PFOs as compared to medical therapy alone.

An echocardiogram with bubbles or with contrast is the main diagnostic tool for a PFO. In a bubble study, agitated saline is first injected into the body during the study. The study is considered positive when contrast is noted to be in the left atrium during early ventricular systole. Contrast effects may be augmented by instructing the patient to cough or perform a Valsalva maneuver, both increasing the sensitivity of this study [2]. A transesophageal echocardiogram (TEE) is then performed in order to both confirm the presence of a PFO and further characterize its anatomy including rim thickness and overall suitability of device closure. This is also performed to rule out other causes of cardioembolic strokes, such as vegetations, mass, or thrombus. Since a PFO can be found incidentally on an echocardiogram, further imaging such as CT angiogram (CTA), MRI brain, EKG, or cardiac monitor may be warranted to rule out other causes of stroke.

Fibroelastomas are the second most common primary cardiac tumor. They generally occur on the aortic valve and may act as a nidus for thrombus formation, thereby posing a risk factor for cardiac embolization systemically, including cerebral destinations. We report a case of a young female who presented with CVA and was ultimately found to have both a PFO and a fibroelastoma on her aortic valve.

## Case presentation

The patient was a 31-year-old Caucasian female with a history of severe migraines who presented to our facility for sudden-onset slurred speech, left-sided weakness, and facial droop for two hours. She was found to have a right middle cerebellar artery (MCA) hyperdensity suspicious for thrombus on CT of the head and was given tissue plasminogen activator (tPA). CTA head and neck showed a proximal M1 occlusion with a large perfusion defect. The findings were discussed with interventional radiology (IR) who performed a mechanical thrombectomy with the removal of a thrombus that was about 0.8 cm in length. Once the patient was stabilized, history elicited that she had previously carried two pregnancies to term but had also experienced a previous miscarriage at 10 weeks gestation. She denied tobacco use and did not take any oral contraceptive pills (OCPs), although she did have a progestin intrauterine device (IUD). Her family history was notable for two miscarriages experienced by her mother.

Her vital signs were stable. On physical exam, she had a National Institutes of Health (NIH) stroke scale score of 0. Her cardiovascular exam was negative for any murmurs, rubs, or gallops. In addition, she had a brisk capillary refill and exhibited no signs of jugular venous distention. Routine labs were remarkable for a low-density lipoprotein (LDL) of 118 mg/dl (normal: <100 mg/dl). A hypercoagulable panel was pertinent for low protein S antigen levels of 53% (normal: 60-150%) and a free protein S level of 55% (normal: 57-157%). In addition, she underwent a workup to rule out antiphospholipid syndrome (APS), which included lupus anticoagulant, anticardiolipin antibodies: immunoglobulin M (IgM) and immunoglobulin G (IgG); and beta-2 glycoprotein antibody: immunoglobulin G (IgG). The APS labs were unremarkable.

**Figure 1:**
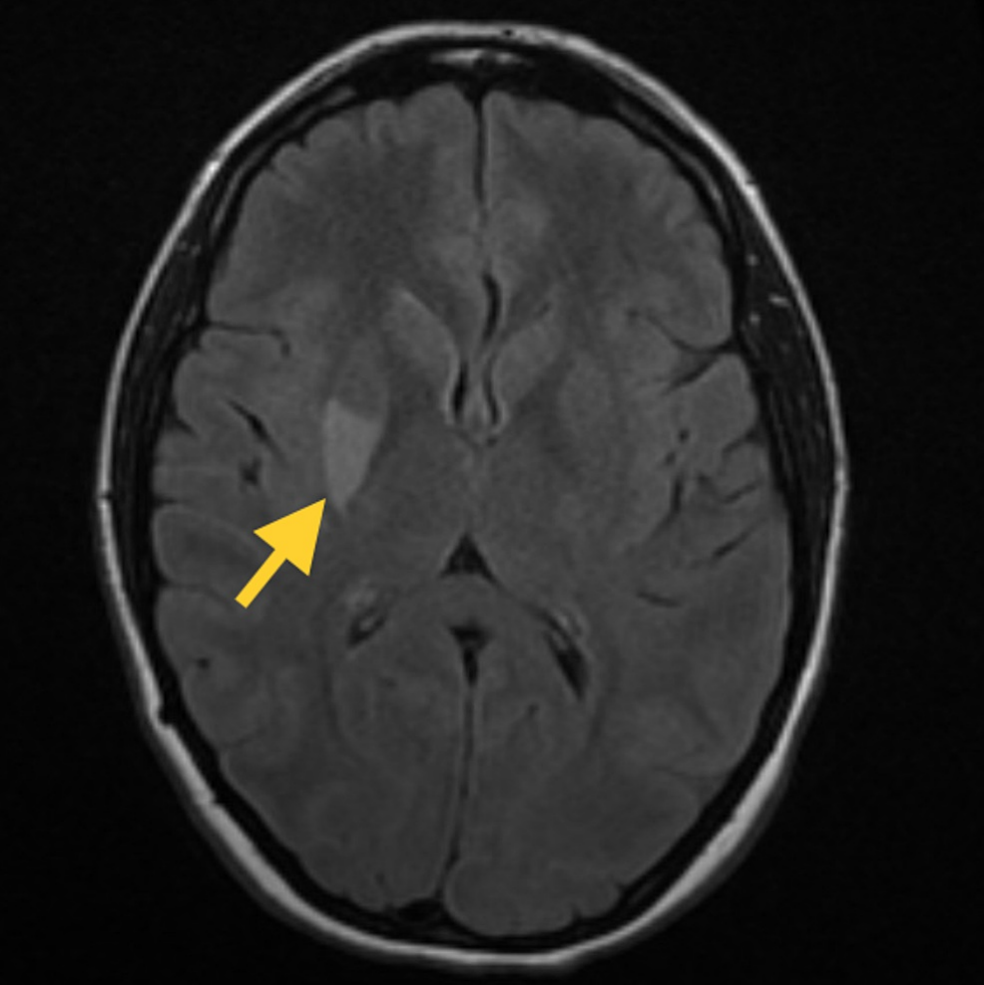
MRI Axial T2 FLAIR demonstrating right insular CVA (yellow arrow) MRI: magnetic resonance imaging; FLAIR: fluid-attenuated inversion recovery; CVA: cerebrovascular accident

**Figure 2:**
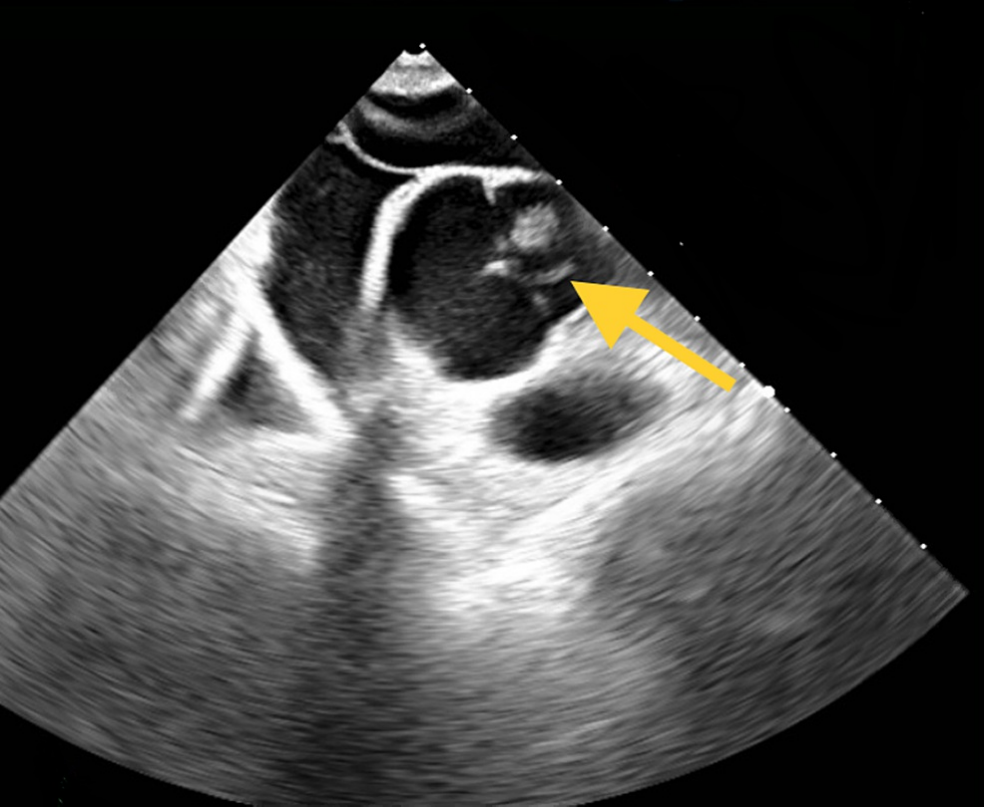
TEE showing 7-mm presumed fibroelastoma present on the aortic valve (yellow arrow) TEE: transesophageal echocardiogram

The EKG showed normal sinus rhythm. Further workup with lower bilateral extremity Dopplers did not show any culprit deep vein thrombosis (DVT). An MRI of the brain showed multiple infarcts in the right basal ganglia, caudate nucleus, and right insula (Figure 1). A transthoracic echocardiogram (TTE) with contrast revealed a PFO with a right to left shunt. A TEE confirmed the prior finding and revealed a 7-mm presumed fibroelastoma on the aortic valve (Figure 2). She had a normal ejection fraction on both echocardiograms. Her Risk of Paradoxical Embolism (RoPE) score index was 9, indicating an 88% attributable fraction and a 2% risk of a two-year recurrence of CVA or transient ischemic attack (TIA).

Cardiothoracic surgery was consulted and they recommended elective PFO closure and resection of the fibroelastoma in one month to allow for adequate healing from the CVA.

The patient was discharged on apixaban for presumed venous thrombosis and instructed to follow up with a hematologist in an outpatient setting. Unfortunately, she developed a subsequent TIA three weeks later, prompting earlier closure of the PFO. A repeat TEE was performed prior to the surgery and demonstrated a PFO on the aortic valve; however, the presumed fibroelastoma compared to the initial ultrasonography was not appreciated. A cardiac MRI was considered, but given the rapid recurrent presentation of her symptoms, the specialists wanted to prevent any further delay. During the transcatheter repair of her PFO, the surgeon was not able to appreciate any residual mass on the aortic valve, throwing into question the role of the presumed fibroelastoma in her CVA. She was discharged with apixaban for three months, after which she was transitioned to aspirin, to be taken indefinitely. Her postoperative course has been unremarkable so far.

## Discussion

PFOs are generally asymptomatic. Some patients develop severe migraines or CVAs, both of which have been shown to improve with PFO closure. The CLOSE trial was a randomized clinical trial that assessed the superiority of PFO closure with antithrombotic therapy as opposed to antithrombotic therapy alone in migraine cessation as well as stroke prevention. The trial concluded that there was no significant difference between the two groups with regard to migraine cessation [2]. Regarding stroke prevention, the CLOSE trial did find PFO closure to be superior to antithrombotic therapy in patients with an initial cryptogenic stroke [3].

Candidates for PFO closure are determined based on the likelihood that an existent PFO is related to the CVA. The RoPE score index was developed to aid in determining the likelihood of a CVA being related to a PFO [4]. The scores range from 1 to 10, with higher numbers indicating a higher likelihood of association. In the study, various clinical factors were considered, including high-risk echocardiogram findings, as well as radiographic findings of stroke, and the association with the risk of CVA in PFO. Significant clinical associations included young age and the absence of diabetes, hypertension, smoking, or prior CVA or TIA (Tables 1, 2). One study found that the risk of stroke increased from 23% [95% confidence interval (CI): 19-26%] in those with 0-3 points to 73% (95% CI: 66-79%) in those with 9 or 10 points [5]. A score of >7 was found to be the most indicative of a CVA being attributed to a PFO.

**Table 1:** RoPE index for the risk of CVA in PFO* *[4] RoPE: Risk of Paradoxical Embolism; CVA: cerebrovascular accident; PFO: patent foramen ovale; TIA: transient ischemic attack

RoPE index
No history of hypertension (1 point)
No history of diabetes (1 point)
No history of CVA or TIA (1 point)
No smoking history (1 point)
Cortical infarct on imaging (1 point)
Age 18-29 years (5 points)
Age 30-39 years (4 points)
Age 40-49 years (3 points)
Age 50-59 years (2 points)
Age 60-69 years (1 point)
Age <70 years (0 points)

**Table 2:** RoPE scoring and probability that CVA is attributable to PFO, as well as the estimated risk of CVA/TIA recurrence in two years* *[5] RoPE: Risk of Paradoxical Embolism; CVA: cerebrovascular accident; PFO: patent foramen ovale; TIA: transient ischemic attack

RoPE score	PFO-attributable fraction (95% CI)	Estimated stroke/TIA recurrence in two years
0–3	0% (0–4)	20% (12–28)
4	38% (25–48)	12% (6–18)
5	34% (21–45)	7% (3–11)
6	62% (54–68)	8% (4–12)
7	72% (66–76)	6% (2–10)
8	84% (79–87)	6% (2–10)
9–10	88% (83–91)	2% (0–4)

An echocardiogram with bubbles or with contrast is the main diagnostic tool for a PFO. In a bubble study, agitated saline is first injected into the body during the study. The study is deemed positive when contrast is noted to be in the left atrium during early ventricular systole. Contrast effects may be enhanced by instructing the patient to cough or perform a Valsalva maneuver, both increasing the sensitivity of this study [6]. A TEE is then performed in order to both confirm the presence of a PFO and further characterize its anatomy including rim thickness and overall suitability of device closure. This also serves to rule out other causes of cardioembolic strokes, such as vegetation, mass, or thrombus. Since a PFO can be found incidentally on an echocardiogram, further imaging such as CTA, MRI brain, EKG, or cardiac monitor may be warranted to exclude other causes of stroke [7].

The treatment of PFO depends on the RoPE score and ruling out cryptogenic stroke. In patients with a low RoPE index (<7), medical therapy with antithrombotic therapy alone may be sufficient if no other high-risk features such as a hypercoagulable state or current DVTs are found. In patients with a high RoPE index (>7), PFO closure and antithrombotic therapy may be a more reasonable option. In patients with concomitant venous thromboembolism (VTE), anticoagulation (such as Coumadin or apixaban) is warranted. After appropriate treatment of VTE, the anticoagulation may be discontinued and antiplatelet therapy is initiated instead. However, if the VTE is determined to be idiopathic, anticoagulation should be continued. 

Our patient had a RoPE index score of 8, which is on the higher end of the spectrum. The patient was negative for any concomitant VTE. However, given her high RoPE score, she was placed on apixaban. Her apixaban was discontinued at three months following the negative results of her hypercoagulable workup and she was continued only on aspirin.

A fibroelastoma, which is the second most common primary cardiac tumor, may complicate the accuracy of scoring systems such as the RoPE [8]. A fibroelastoma may act as a nidus for thrombi. In addition, the typical location of fibroelastoma is on the aortic valve, which may pose a risk factor for cardiac embolization systemically, including cerebral destinations [9].

Open procedures are pursued if there are concomitant abnormalities in the heart requiring surgical intervention. However, a less invasive transcatheter approach is generally utilized in the absence of other cardiac abnormalities. The catheter is advanced through the femoral vein bypassing the right atrium, through the PFO, and into the left atrium. Next, a PFO closure device is deployed to straddle the two sides of the heart surrounding the defect (i.e., the right and left atrium). The catheter is then removed and the procedure is complete.

Contraindications to PFO closure include patients with a pre-existing inferior vena cava (IVC) filter, bleeding diathesis, thrombophilia, or unsuitable anatomy. Even this minimally invasive percutaneous procedure has its risks. Potential complications include arrhythmias, device migration or embolization, device erosion, traumatic puncture of coronary vessels with resulting hemorrhage, infection, and paradoxically, CVA. The most common arrhythmia following closure is new-onset atrial fibrillation or atrial flutter, which can be attributed to the location of the closure device [10]. Post-procedure, patients are typically first treated with aspirin and clopidogrel for three months, following which clopidogrel is stopped and aspirin is continued.

Since our patient had no further recurrence of stroke following the PFO closure, we consider the PFO as an independent risk factor for her initial episode of stroke. Concomitant PFO and cardiac tumors are rare with only eight reported cases in the literature so far [11]. In those cases, PFO and cardiac tumors may have acted synergistically to cause a cardioembolic stroke.

The fibroelastoma on our patient’s initial TTE and TEE was not appreciated on subsequent TEE where her cardiologist presumed that the tumor may have embolized. Embolization of fibroelastoma inducing cardioembolic stroke can occur in two of the following ways: either through the fragmentation of the tumor itself or from the formation of thrombi on the surface of the tumor [12]. According to a study by Gowda et al., cases of fibroelastoma embolization manifest as a TIA or stroke in 17% of instances [13].

Our patient had no obvious signs of masses or vegetations intraoperatively, and hence there were no available histopathologic specimens to confirm the diagnosis of fibroelastoma. In addition, her head imaging was negative for any new infarcts or large vessel occlusion (LVO). Perhaps, if the patient’s symptoms of stroke had not reoccurred so rapidly, a cardiac MRI might have been able to be performed prior to the surgery. According to a study by Sloimsky et al., a cardiac MRI has a sensitivity of 98% and specificity of 86.6% for diagnosing cardiac tumors. Furthermore, their study recommends that cardiac MRI be included in the routine evaluation of cardiac masses, especially when preliminary imaging studies are inconclusive [14].

## Conclusions

The treatment of CVA in a patient with a PFO requires a risk-driven approach. Risk stratification scores such as the RoPE index have been shown to accurately predict the likelihood that a CVA is associated with a PFO. It can be calculated in order to help physicians decide whether to pursue surgical intervention or not. Medical therapy alone using antiplatelet therapy may be sufficient in patients with low-risk scores. However, in high-risk patients with a RoPE score >7, a PFO closure may be warranted. A hypercoagulable workup should be considered in patients suspected of having thrombophilia, in which anticoagulation in addition to surgery is indicated. A cardiac MRI may be warranted prior to the surgery in cases with conflicting preliminary studies to determine the presence of a cardiac tumor.
